# Case Report: long-term survival of a male patient with breast cancer complicated by lung adenocarcinoma treated with individualized therapy

**DOI:** 10.3389/fonc.2026.1780512

**Published:** 2026-02-25

**Authors:** Yadong Liu, Xuejuan Duan, Zhenru Zheng, Gang Cheng, Xianbo Zhang, Jing Zhao

**Affiliations:** 1 Department of Oncology, Hebei General Hospital, Shijiazhuang, China; 2 Department of Radiation Oncology, The Fourth Hospital of Hebei Medical University, Shijiazhuang, China

**Keywords:** invasive ductal carcinoma, lung adenocarcinoma, male breast cancer, multiple primary malignancies, second primary malignant tumor

## Abstract

Male breast cancer (MBC) is a rare malignant tumor with unique clinical characteristics, accounting for only approximately 1% of all breast cancer cases. Although its pathological features are similar to those of female breast cancer (FBC), significant differences exist in the clinical manifestations, diagnosis, and prognosis of MBC. This article reports a case of a male patient with stage IV lung cancer complicated by invasive ductal carcinoma of the breast, which is extremely rare in MBC. The particularity of this case lies in the sequential occurrence of two malignant tumors, highlighting the importance of early identification of MBC to improve the overall prognosis of patients. Through detailed analysis of this case, we found that the potential pathogenic mechanism of MBC needs further investigation, and clinicians must maintain a high degree of vigilance when evaluating patients with existing malignant tumors to promptly identify potential coexisting tumors. We call for enhanced awareness of MBC in clinical practice to achieve early diagnosis and treatment, thereby improving patient survival rates.

## Introduction

Male breast cancer (MBC) is a rare malignant tumor, accounting for approximately 1% of all breast cancers. Its clinical characteristics, diagnosis, and prognosis are significantly different from those of female breast cancer (1, 2). The incidence of second primary malignant tumors in long-term cancer survivors is gradually increasing, especially after advanced lung cancer patients achieve long-term survival through effective treatment, the risk of secondary other malignant tumors requires attention (3, 4). However, cases of secondary male breast cancer as a second primary malignant tumor following lung adenocarcinoma (especially in stage IV patients) are extremely rare, and there is currently a lack of relevant clinical data to support diagnosis and treatment decisions.

The core clinical value of this case lies in: 1) It is the first detailed report on the clinical characteristics of secondary male breast cancer in long-term survival patients with stage IV lung adenocarcinoma, providing a typical case for the clinical identification of second primary malignant tumors; 2) It emphasizes that the diagnosis of male breast cancer is prone to delay due to gender bias. For male patients with a history of malignant tumors, the potential malignant risk of breast masses should be vigilant during follow-up; 3) The achievement of a progression-free survival period of more than 32 months through individualized treatment provides a practical basis for the formulation of treatment plans for similar rare cases. Based on this, this study reports the case and combines a literature review to explore its diagnostic points, treatment strategies, and clinical significance, in order to improve clinicians’ understanding of this type of disease.

## Case presentation

**Figure 1:**
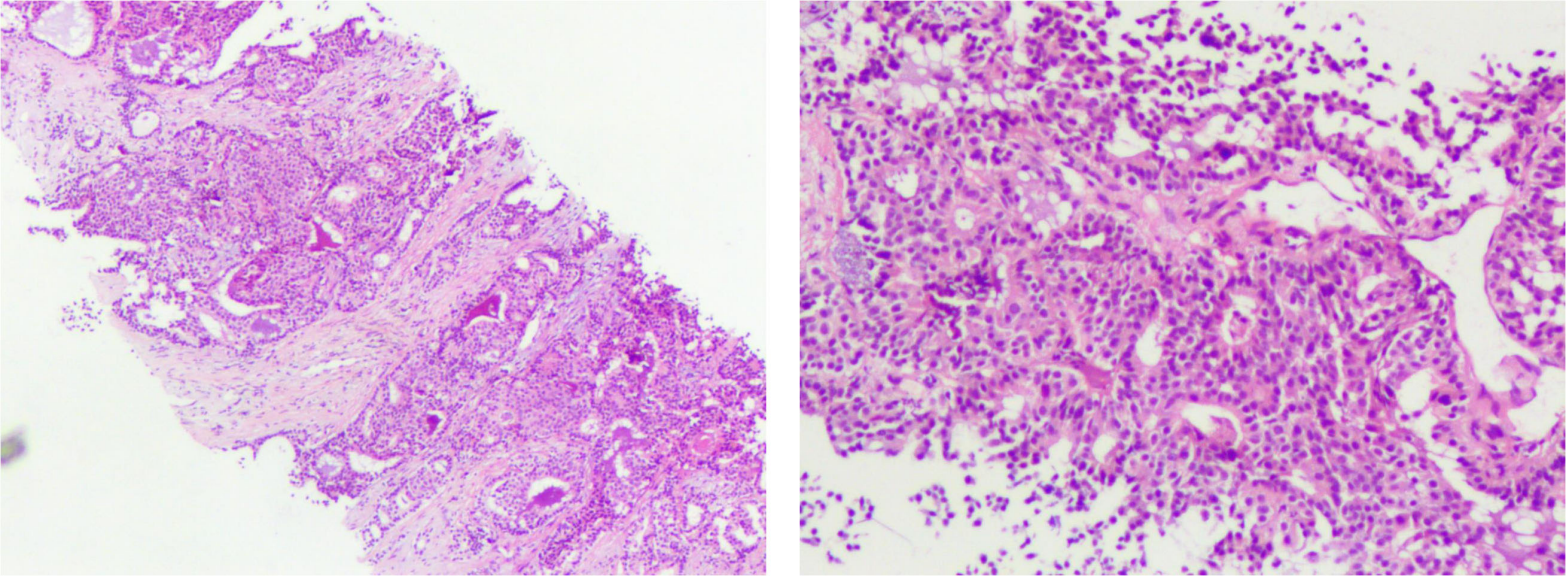
Pathological image of lung cancer. **(A)** Hematoxylin and eosin (H&E) stain, ×40 magnification. **(B)** Hematoxylin and eosin (H&E) stain, ×200 magnification.

This case report describes a male patient who was initially diagnosed with right lung cancer in 2019(**Supplementary Materials**). Clinical stage (cTNM): cT1cN3M1a, stage IVA (the maximum diameter of the tumor is approximately 2.1 cm; chest CT indicates mediastinal lymph node metastasis, bilateral hilar lymph node metastasis, and bilateral lung metastasis). Pathological examination of the needle biopsy specimen confirmed invasive adenocarcinoma (**Figure 1**). No common gene mutations, including EGFR and ALK, were detected at the time of diagnosis. The patient received 8 cycles of chemotherapy with pemetrexed plus lobaplatin. After treatment, severe myelosuppression occurred (grade III neutropenia with fever). Considering that the patient is an advanced cancer survivor with a limited expected survival time, after a comprehensive evaluation by the multidisciplinary team (MDT), it was deemed that the benefits of further chemotherapy were less than the risk of toxicity. Therefore, adjuvant chemotherapy was not administered, and no additional chemotherapy drugs were used. achieving a partial response (PR), and subsequent regular follow-up showed stable disease. A follow-up examination in March 2023 revealed that the intrapulmonary metastatic lesions had enlarged compared with prior images; radiofrequency ablation was subsequently performed for the intrapulmonary metastases, and the patient’s condition remained stable on subsequent follow-ups.

**Figure 2:**
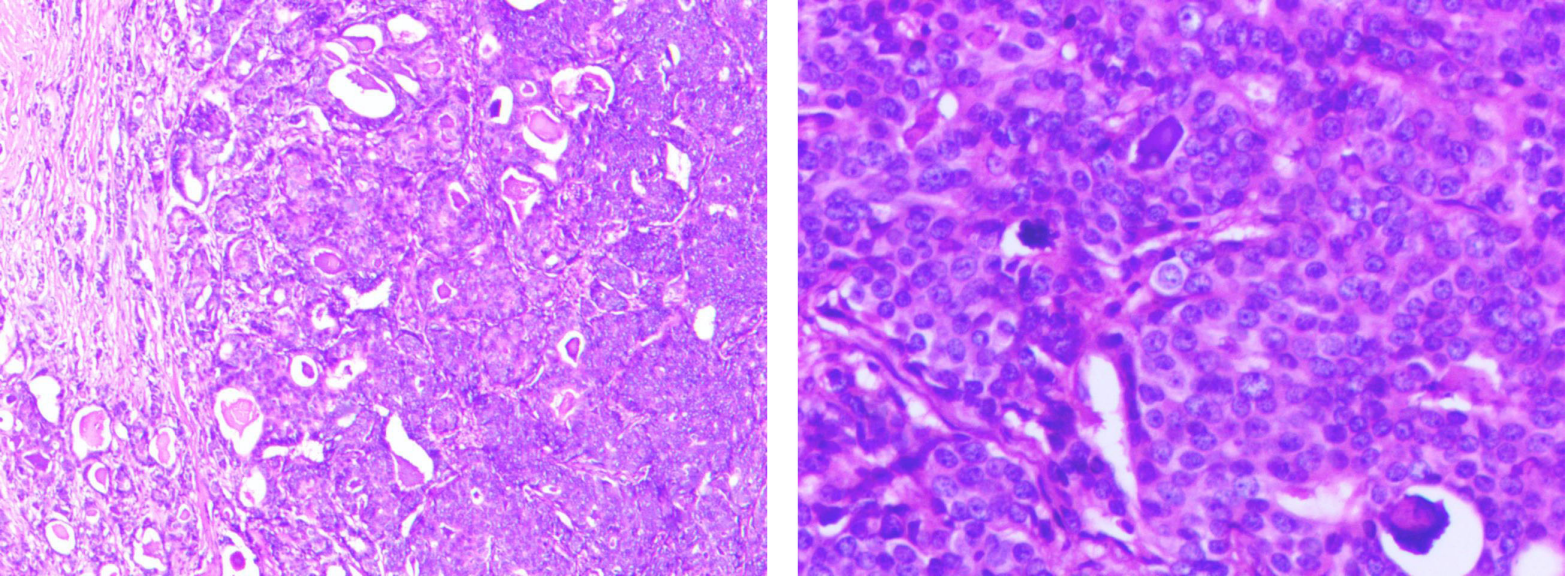
Pathological image of breast cancer. **(A)** Hematoxylin and eosin (H&E) stain, ×100 magnification. **(B)** Hematoxylin and eosin (H&E) stain, ×200 magnification.

On April 6, 2023, a right breast mass was detected during follow-up. The patient presented to the local county hospital and was misdiagnosed with a simple subcutaneous mass. A mass resection and biopsy was performed on April 7, 2023. Postoperative pathological examination on April 12, 2023, indicated breast cancer, with the tumor measuring 1.6 cm × 1.6 cm × 1.0 cm (relevant imaging data were unavailable). The patient was transferred to our hospital on April 16, 2023. Ultrasonography revealed postoperative changes at the right breast mass resection site, and a hypoechoic nodule in the subcutaneous soft tissue at the 9 o’clock position along the anterior axillary line of the right chest wall, with a size of approximately 9.4 mm × 6.5 mm (**Supplementary Materials**). No obvious axillary lymphadenopathy was observed. For the purpose of radical resection. On April 17, 2023, the patient underwent “modified radical mastectomy for right breast cancer + sentinel lymph node biopsy” under general anesthesia. Intraoperatively, sentinel lymph nodes (SLNs) were identified using a tracer method, and a total of 9 SLNs were detected (2 of which were positive on intraoperative frozen section). Given the positive sentinel lymph node metastasis and the patient’s small breast tissue volume, to thoroughly eliminate potential metastatic lymph nodes and reduce the risk of local recurrence, axillary lymph node dissection (ALND) was promptly converted from SLN biopsy. Postoperative pathological results (**Figure 2**) showed: (right breast tumor, size 1.5 cm * 1.4 cm * 1.0 cm) invasive ductal carcinoma Grade II, with perineural invasion but no definite lymphovascular invasion. No carcinoma was found in the nipple, skin, or basal soft tissue. Regional lymph node metastasis was detected in 2 out of 24 nodes, grouped as follows: (sentinel lymph nodes) 2/9 (combined with frozen section); (Level I;) 0/13; (Level II) 0/2; (Level III) no carcinoma in soft tissue. Postoperative staging: pT1cN1aM0, stage IIA.

**Figure 3:**
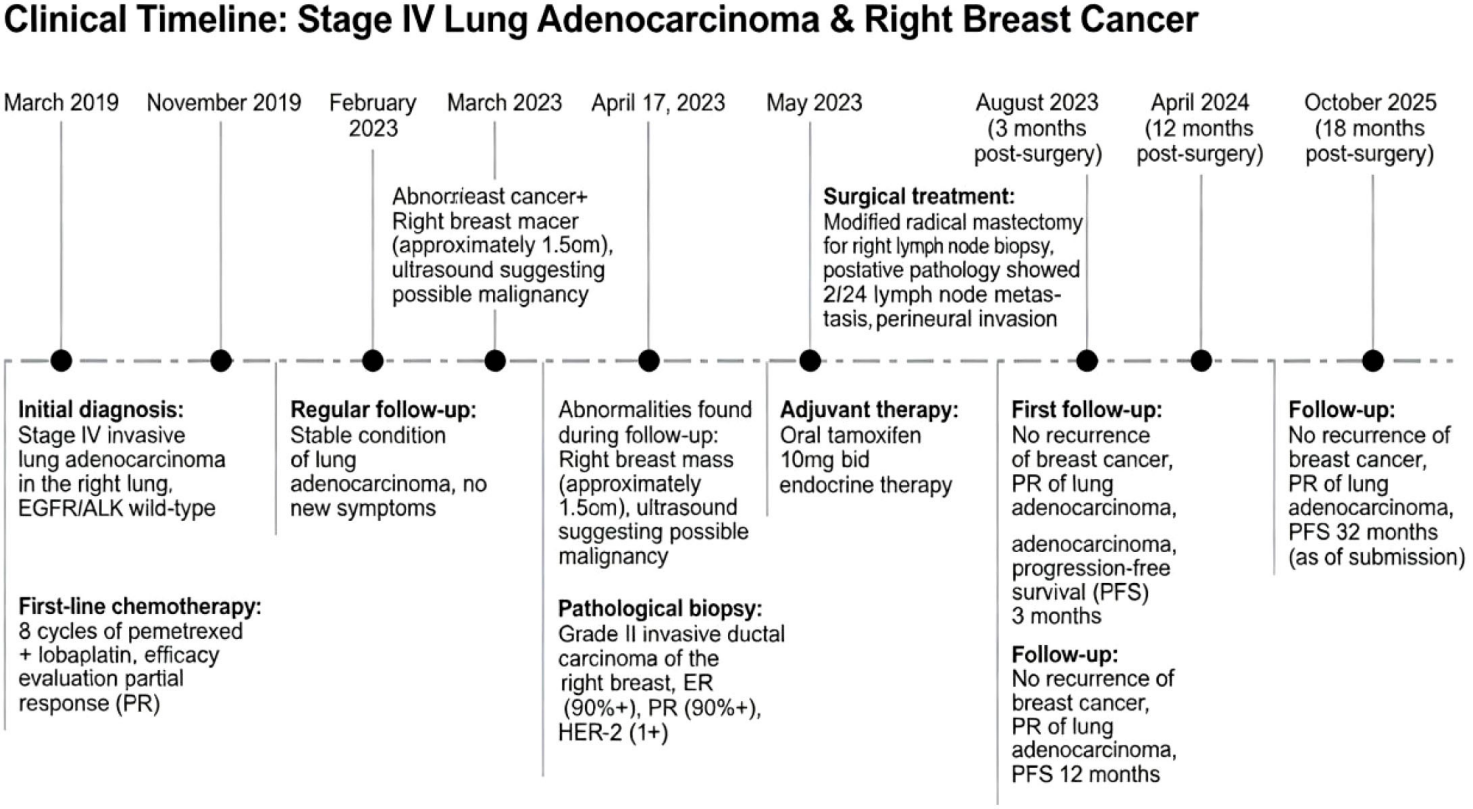
Key timelines for treatment and follow-up.

Immunohistochemical staining results: M23-B2: ER (approximately 90%, moderate-strong +), PR (approximately 90%, moderate-strong +), HER-2 (1+), Ki-67 (approximately 15% +), P53 (-), CK5/6 (-), TOPOII (Grade II), E-cadherin (+), AR (approximately 60%, weak +), P63 (-), Calponin (-). M23-A: HER-2 (0). Based on the patient’s pathological results and breast cancer guidelines, radiotherapy, endocrine therapy, and genetic testing were recommended to determine the need for chemotherapy. Considering the particularity of this case (male lung adenocarcinoma patient with second primary breast cancer), a multidisciplinary team (MDT) consultation was conducted, including specialists from breast surgery, pathology, medical oncology, imaging, and radiation oncology. Comprehensive consideration of various factors and the patient’s specific condition led to the decision of endocrine therapy (tamoxifen 10mg twice daily, oral administration). Regular follow-up showed no local recurrence or distant metastasis after surgical resection of breast cancer, and the efficacy evaluation of lung cancer was partial response (PR). (**Supplementary Materials**). As of submission, the patient’s progression-free survival time after diagnosis of breast cancer has exceeded 32 months(**Figure 3**).

## Discussion

Male breast cancer (MBC) is a rare malignant tumor with unique clinical characteristics, and its incidence is significantly lower than that of female breast cancer (FBC) (1, 2). This case presents a male patient with previously diagnosed stage IV lung cancer who was subsequently found to have concurrent invasive ductal carcinoma of the breast. Male breast cancer as a second primary malignant tumor secondary to lung adenocarcinoma is rarely reported in the literature, which is confirmed by a systematic search of PubMed, Embase, Web of Science, and Cochrane Library from database establishment to date.

Literature indicates that male patients are usually diagnosed around 60 years of age, with typical manifestations of a painless breast mass, which may be accompanied by local lymphadenopathy. In the diagnosis of MBC, misdiagnosis or missed diagnosis is common due to atypical symptoms, and doctors’ gender bias may further exacerbate this problem, leading to poor treatment outcomes (5, 6). The incidence rate of second primary malignant tumors in long-term cancer survivors is approximately 5%-10% (3). Among them, the risk of lung cancer survivors developing other solid tumors is 2–3 times higher than that of the general population, while the incidence rate of male breast cancer as a second primary cancer is less than 0.1%. Studies have shown that the clinical manifestations of MBC differ from those of FBC; male patients usually present with larger tumors at the time of onset and are more prone to lymph node metastasis, which may be related to higher estrogen levels and other biological factors in males (7, 8).

The pathogenesis of MBC is not yet fully understood, but it may be related to sex hormone levels, genetic factors, and environmental exposure (3). Scholars have pointed out that the incidence of MBC is significantly higher in certain special populations than in the general population. For example, the incidence of breast cancer in carriers of BRCA2 gene mutations is as high as 7.1% (9). In addition, the incidence of MBC is also higher in veterans, especially among prostate cancer survivors (10). Moreover, studies have shown that MBC patients may have a higher response rate to endocrine therapy, which is closely related to the hormone receptor status of their tumors (11). In this case, invasive ductal carcinoma showed high invasiveness in a male patient, and the immunohistochemical results (ER and PR positive, HER-2 negative) provided a basis for subsequent treatment.

In terms of the treatment of MBC, the main method is modified radical mastectomy, but there are certain differences in surgical selection between MBC and FBC. Studies have shown that the application rate of modified radical mastectomy in MBC patients (64%) is similar to that in FBC patients (61%), with no statistically significant difference. However, due to the often larger tumors at diagnosis and less breast tissue in males, the application rate of breast-conserving surgery (BCS) in MBC is lower. In addition, insufficient awareness of breast cancer in males may lead to delayed treatment, thereby affecting the choice of surgical timing. Therefore, although the surgical methods are similar, surgical treatment of MBC requires more emphasis on early diagnosis and individualized plans, especially for patients with previous malignant tumors.

For this patient, since only mass resection and biopsy were performed in the initial surgery and the patient was complicated with another mass in the right chest wall, right modified radical mastectomy combined with sentinel lymph node biopsy was planned for radical resection, despite the absence of enlarged lymph nodes on axillary examination. Intraoperatively, frozen pathological examination of the biopsied lymph nodes indicated metastasis in 2 nodes; therefore, modified radical mastectomy plus axillary lymph node dissection was finally performed. However, there is no clear consensus on adjuvant treatment regimens. Due to the lack of large-sample clinical studies, some studies suggest that experience from FBC can be used for reference (12).Referring to FBC treatment guidelines, adjuvant radiotherapy, chemotherapy, and endocrine therapy should be administered after surgery. However, after a multidisciplinary consultation, considering that the patient’s postoperative pathology indicated 2/24 regional lymph node metastasis and perineural invasion, which suggested potential indications for adjuvant radiotherapy. However, combined with the patient’s chest CT showing that the lung adenocarcinoma lesions were still in a partial response (PR) state, chest radiotherapy might cause superimposed damage to lung function. Additionally, the patient was older (67 years old) with generally reserved basic lung function. After MDT discussion, it was considered that the local control benefit brought by radiotherapy was not clearly superior to the risk of lung function damage. Eventually, adjuvant radiotherapy was not performed, and no radiotherapy plan was formulated or implemented. Comprehensive consideration led to the decision of oral tamoxifen endocrine therapy, and the patient has achieved a PFS of 32 months to date. The treatment decision for this case is an individualized choice and does not mean that all similar cases do not require radiotherapy or chemotherapy. Clinically, a comprehensive judgment should be made based on the patient’s previous treatment history, physical condition, and biological characteristics of the tumor.

Germline mutations in the BRCA2 gene are an important genetic risk factor for male breast cancer. The incidence of breast cancer in men carrying this mutation is as high as 7.1% (9). Moreover, patients with BRCA mutations may be more sensitive to platinum-based chemotherapy and PARP inhibitors. Therefore, it is recommended that male breast cancer patients undergo routine germline testing for BRCA1/2 genes (13). In this case, the patient and his family refused genetic testing due to economic reasons, so data on the BRCA gene status were not obtained, which is one of the limitations of this study. Considering that this case is a second primary malignant tumor, BRCA mutation may be a common pathogenic factor for the double primary cancers, but there is a lack of direct evidence to support this. For similar cases in the future, routine germline testing for BRCA genes should be carried out to provide a basis for exploring the etiology and optimizing treatment plans.

## Conclusion

Through this case, we conducted in-depth thinking on the uniqueness of MBC and emphasized the key points in its clinical diagnosis and treatment. The particularity of this case not only lies in being the first report of MBC concurrent with lung cancer in a male patient but also in revealing the clinical challenges of MBC as a second primary malignant tumor. The rarity and atypical clinical manifestations of MBC often lead to its neglect in the diagnostic process. Doctors’ gender bias and insufficient vigilance towards male breast lesions are important factors leading to misdiagnosis and missed diagnosis. Therefore, clinicians need to improve their understanding of MBC, especially maintaining a high degree of vigilance towards breast masses in patients with a history of multiple tumors. This case provides a new perspective for the early identification and diagnosis of MBC, emphasizing the importance of MDT collaboration and individualized treatment to improve patients’ survival rates and quality of life.

Due to the particularity of this case, the generalizability and applicability of the results may be limited. Furthermore, the lack of imaging data from the patient’s initial resection and biopsy for breast cancer has also compromised the completeness of this medical record. Future research should focus on larger sample sizes to verify the findings of this case. Meanwhile, the pathological mechanism of MBC is not yet fully clear, and future studies should further explore its biological characteristics and differences in clinical manifestations to develop more effective treatment strategies.

## Data Availability

The original contributions presented in the study are included in the article/**Supplementary Material**. Further inquiries can be directed to the corresponding author.

## References

[1] Wang Y Chen K Yang Y Tan L Chen L Zhu L . Incidence and survival outcomes of early male breast cancer: a population-based comparison with early female breast cancer. Ann Transl Med. (2019) 7:536. doi: 10.21037/atm.2019.10.04, PMID: 31807518 PMC6861739

[2] Lin AP Huang TW Tam KW . Treatment of male breast cancer: meta-analysis of real-world evidence. Br J Surg. (2021) 108:1034–42. doi: 10.1093/bjs/znab279, PMID: 34476472

[3] Salhab RQ Ghazaleh ZI Barbarawi W Salah-Aldin R Hour H Sweity R . An unusual occurrence of multiple primary Malignant neoplasms: a case report and narrative review. Front Oncol. (2024) 14:1381532. doi: 10.3389/fonc.2024.1381532, PMID: 39087028 PMC11288870

[4] Asgharian M Moslemi D Nikbakht HA Jahani MA Bijani A Mehdizadeh H . Male breast cancer: a 32-year retrospective analysis in radiation therapy referral center in northern Iran. Ann Med Surg (Lond). (2024) 86:5756–61. doi: 10.1097/MS9.0000000000002571, PMID: 39359768 PMC11444606

[5] Lee J Kim KE Kim MK Kim H Ko ES Ko EY . Impact of adding preoperative magnetic resonance imaging to ultrasonography on male breast cancer survival: a matched analysis with female breast cancer. Ultrasonography. (2025) 44:72–82. doi: 10.14366/usg.24130, PMID: 39523655 PMC11717687

[6] Sun H Zhang L Wang Z Gu D Zhu M Cai Y . Single-cell transcriptome analysis indicates fatty acid metabolism-mediated metastasis and immunosuppression in male breast cancer. Nat Commun. (2023) 14:5590. doi: 10.1038/s41467-023-41318-2, PMID: 37696831 PMC10495415

[7] Qian X Zou X Xiu M Liu Y Chen X Xiao M . Epidemiology and clinicopathologic features of breast cancer in China and the United States. Transl Cancer Res. (2023) 12:1826–35. doi: 10.21037/tcr-22-2799, PMID: 37588736 PMC10425668

[8] Lissidini G Nicosia L Sargenti M Cucchi MC Fabi A Falco G . Male breast cancer: a multicenter study to provide a guide for proper management. Breast Cancer Res Treat. (2024) 208:29–40. doi: 10.1007/s10549-024-07380-0, PMID: 38896332

[9] Badve SB Kim E Sibia US Borrego OT Vara S Damron A . Male breast cancer with dual BRCA2 and BRIP1 deleterious gene mutations. Ochsner J. (2024) 24:157–61. doi: 10.31486/toj.23.0119, PMID: 38912178 PMC11192226

[10] Whyne EZ Choi SH Unni N Kanjwal S Dowell JE Jeon-Slaughter H . Factors associated with male breast cancer incidence among prostate cancer survivors: real world evidence from veterans affairs national prostate cancer data core. Prostate. (2025) 86(2):227–35. doi: 10.1002/pros.70074, PMID: 41063384 PMC12704238

[11] Viehweger F Gusinde J Leege N Tinger LM Gorbokon N Menz A . Estrogen receptor expression in human tumors: A tissue microarray study evaluating more than 18,000 tumors from 149 different entities. Hum Pathol. (2025) 157:105757. doi: 10.1016/j.humpath.2025.105757, PMID: 40054585

[12] Huang K Yu Y Li X Liu Y Huang K Wang X . The Impact of Post Mastectomy Radiotherapy on T1-2N0–1 Male Breast Cancer and establishment of an artificial neural network predicting model: Population-Based Study. Cancer Res Treat. (2025) 5. doi: 10.4143/crt.2025.518, PMID: 41197522

[13] Lee J Lee KS Sim SH Chae H Sohn J Kim GM . Impacts of subtype on clinical feature and outcome of male breast cancer: multicenter study in korea (KCSG BR16-09). Cancer Res Treat. (2023) 55:123–35. doi: 10.4143/crt.2021.1561, PMID: 35344650 PMC9873331

