# Supplementary figures and images for: Case Report: long-term survival of a male patient with breast cancer complicated by lung adenocarcinoma treated with individualized therapy
Source: Front Oncol. 2026 Feb 25;16:1780512. doi: 10.3389/fonc.2026.1780512 (PMC12976741; doi:10.3389/fonc.2026.1780512)

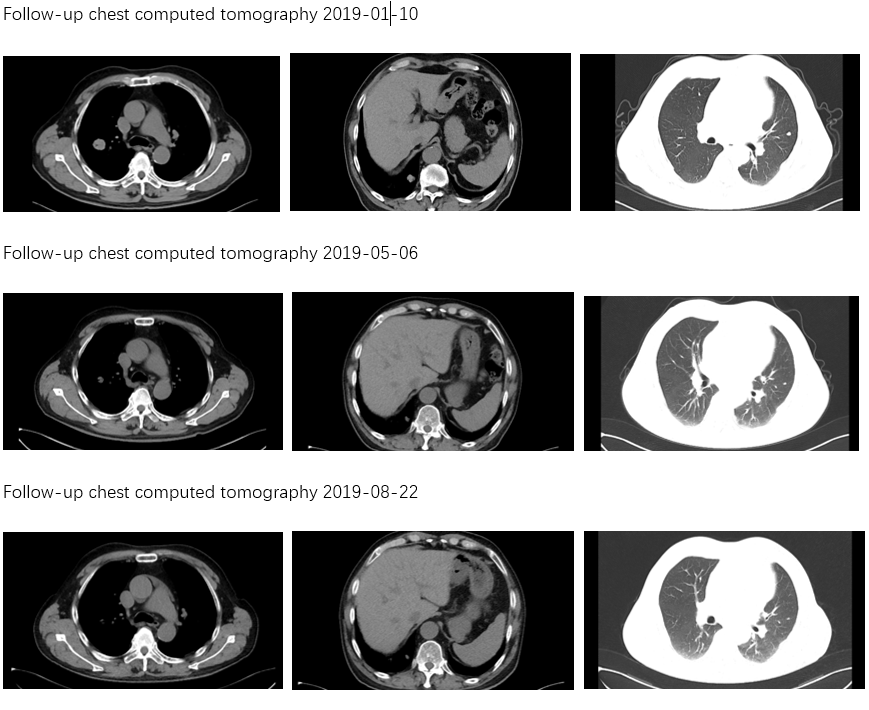

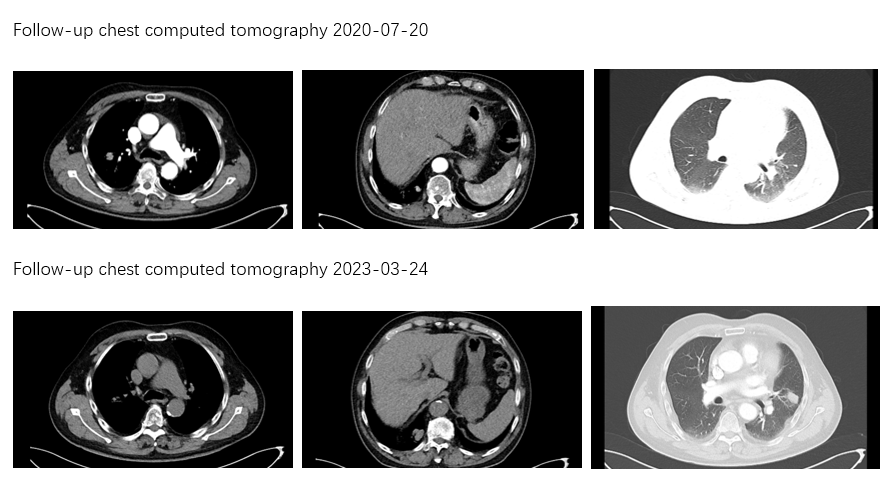

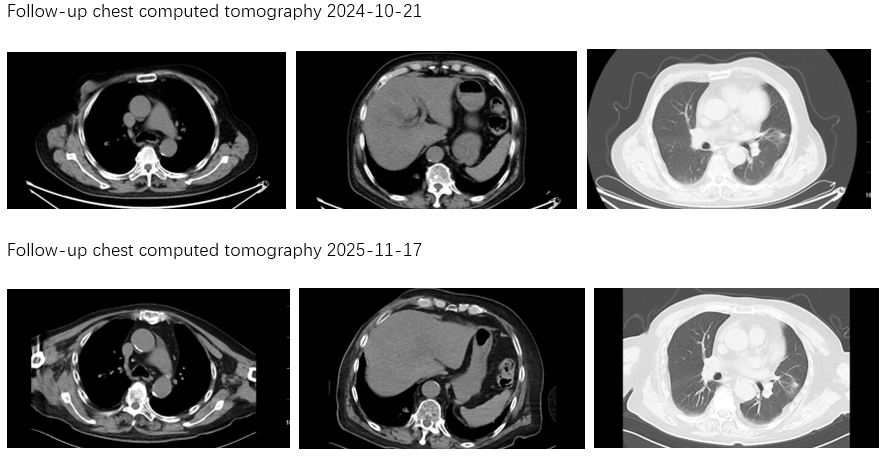

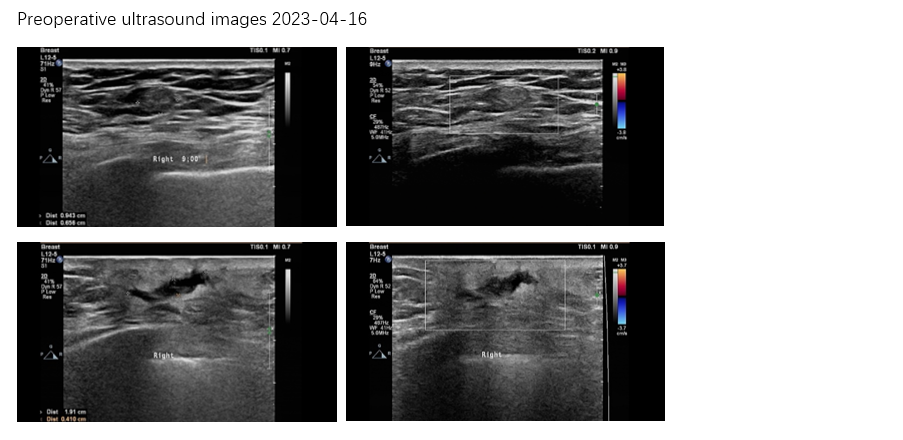

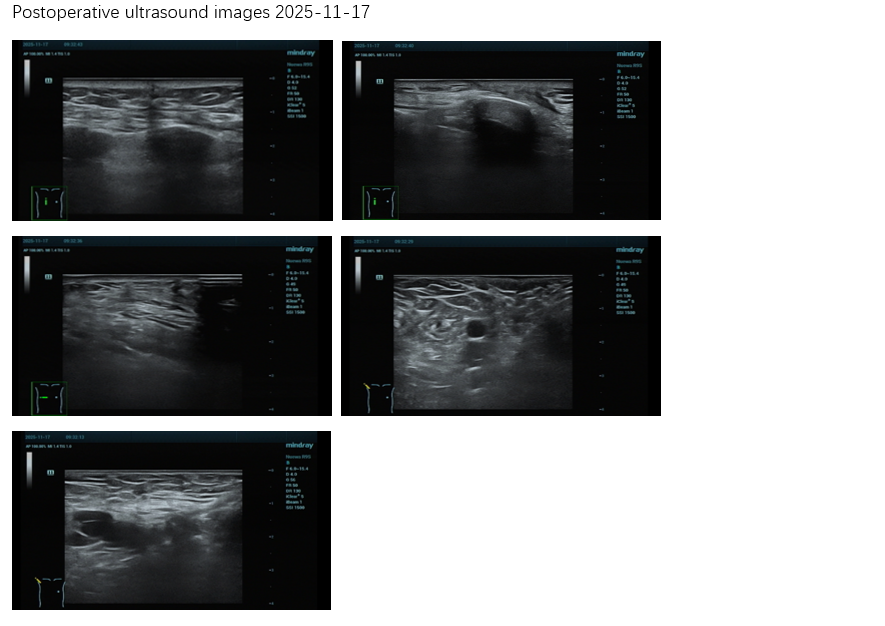

Supplement: Supplementary file 1 [file DataSheet1.docx]
